# Effect of diet on postprandial glycemic and insulin responses in healthy dogs

**DOI:** 10.3389/fvets.2023.1201611

**Published:** 2023-07-18

**Authors:** Alessandro Vastolo, Manuela Gizzarelli, Alessio Ruggiero, Maria Chiara Alterisio, Serena Calabrò, Maria Ferrara, Monica Isabella Cutrignelli

**Affiliations:** Department of Veterinary Medicine and Animal Production, University of Naples Federico II, Naples, Italy

**Keywords:** starch, protein, glucose, fructosamine, insulin, energy

## Abstract

**Introduction:**

Dog owners have gradually changed their approach, paying more attention to the nutrition and health of their animals. Various pet foods with different ingredients and nutritional characteristics are available on the market. The present study aimed to evaluate the administration of three diets, namely, two grain-free (GF1 and GF2) and one grain-based (CB), with different sources of carbohydrates that can influence the glycemic and insulin postprandial responses in healthy dogs.

**Materials:**

Fifteen healthy dogs were dived in three groups and alternatively fed each diet for 50 days. Blood samples were collected at beginning of each feeding period. Glycemia and insulin were measured before and after 120, 240 and 360 minutes diet administration to evaluate postprandial responses.

**Results:**

GF2 diet showed the highest level of albumin and mean insulin concentration (*p* < 0.001). Furthermore, the GF1 diet caused the smallest (*p* < 0.001) glucose and insulin area under the curve (AUC) and the lowest (*p* < 0.05) glucose nadir. Otherwise, GF1 showed the highest (*p* < 0.01) insulin time to peak. The GF2 diet showed the highest level of albumin while reporting the lowest amount of fructosamine (*p* < 0.05). The diet GF2 registered the highest (*p* < 0.001) level of insulin zenith. The cereal-based (CB) diet reported the highest amount of fructosamine (*p* < 0.05). The CB diet had the highest levels of glucose and the highest (*p* < 0.001) glucose and insulin mean concentrations. Diet CB reported the lowest (*p* < 0.001) insulin nadir.

**Discussion:**

Diets with different carbohydrate sources and chemical compositions could modulate the glycemic response in healthy dogs. Bearing in mind that glycemic/insulin postprandial responses influence energy availability and that different dogs have specific lifestyles, it may be preferable to also consider these aspects when choosing a maintenance diet for animals

## 1. Introduction

The role of companion animals in society has undergone great changes over time, especially in large population centers, where they have become indispensable (1). In 2022, the annual report of the Federation of European Pet Food Industries (FEDIAF) reported that 90 million European households own at least one pet. Most pet owners pay special attention to their animals, including their diet. Before the advent of industrial feeds, dogs were often fed kitchen and/or butcher shop scraps. Owners’ approach has gradually changed as new knowledge about companion animal nutrition has developed (2, 3). As a result, a huge amount of pet foods characterized by different ingredients and nutrient concentrations have entered the market over the past two decades. In this regard, the content of carbohydrates (e.g., soluble sugars, starch, and dietary fiber) varies greatly among commercial pet food brands. Over the years, the domestication of dogs has improved their ability to digest and metabolize carbohydrates (4). As indicated by Carciofi et al. (5), starch is known as a palatable and digestible source of energy. In addition, carbohydrates allow dogs to store essential nutrients, such as amino acids or fatty acids, especially during specific stages of life. However, no specific carbohydrate requirements have been indicated for companion animals (2, 3, 6). Intrinsic carbohydrate availability may change owing to variations in protein and fat content and the technological processes used. Furthermore, it has been shown that starch digestibility is highly variable, and it is influenced by several factors, such as sources, particle size, amylose:amylopectin ratio, processing methods (7), and starch:protein ratio (8). In addition, all these factors can affect postprandial glycemic levels in healthy dogs (5, 9, 10). The postprandial glycemic response can be assessed on both single and mixed foods. However, the presence of protein and fat may affect the responses and vary the differences between foods. This study aimed to evaluate whether the administration of three diets, namely, two grain-free diets (GF1 and GF2) versus one cereal-based diet (CB), formulated with different sources and amounts of carbohydrates, can influence the postprandial glycemic response in healthy dogs.

## 2. Materials and methods

### 2.1. Animal and diets

All the procedures used in the study were approved by the Ethics Committee for the Care and Use of Animals of the University of Naples Federico II in accordance with local and national regulations and guidelines (Legislative Decree 26 of 04/03/2014).

**Figure 1:**
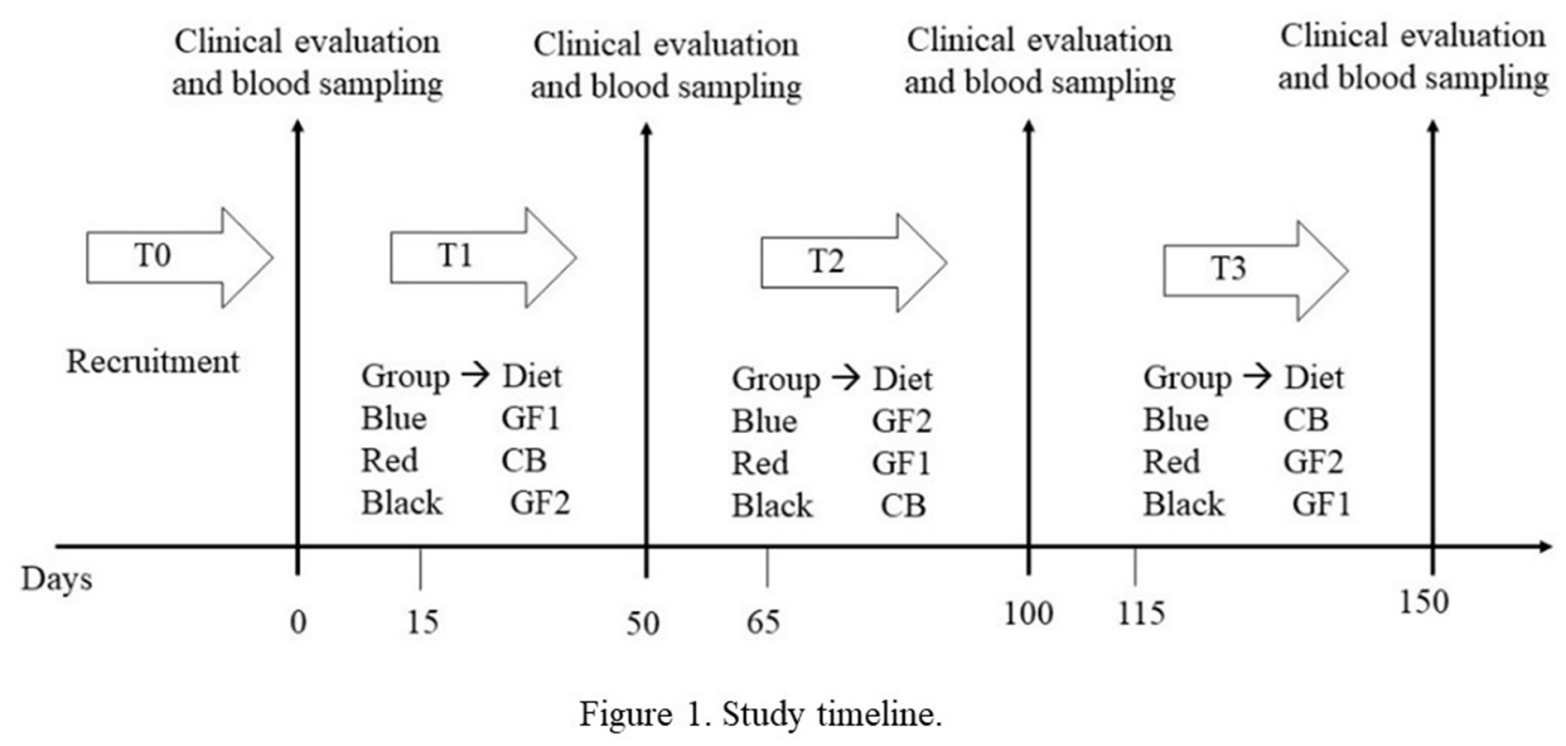
Study timeline.

In all, 15 neutered healthy adult dogs (mean age 5.00 ± 1.30 years, body weight 21.1 ± 5.36 kg, and BCS 4.20 ± 0.86 on 5 points scale) were recruited in a private kennel located in the province of Naples (Italy) and homogeneously divided into three groups, which were alternatively fed with three commercial kibble diets (Figure 1). At the time of recruitment, no clinical signs, clinicopathological changes, or the presence of mainly canine vector-borne diseases were observed.

The diets were formulated using the same main protein source (chicken), but different carbohydrate sources and were named GF1, GF2, and CB. The ingredients, chemical compositions, and essential amino acids of each diet are reported in Tables 1–3, respectively.

**Table 1:** Ingredients of three tested diets.

Diet	Ingredients
GF1	Boneless chicken, dehydrated chicken protein, sweet potato, chicken fat, dried eggs, herring, dehydrated herring protein, fish oil (from herring), pea fiber, and dried carrot.
GF2	Boneless chicken, dehydrated chicken protein, pea starch, chicken fat, dried pumpkin, dried eggs, herring, dehydrated herring protein, fish oil (from herring), pea fiber, and dried carrot.
CB	Boneless chicken, dehydrated chicken protein, spelt, oats, chicken fat, dried eggs, herring, dehydrated herring protein, dried beet pulp, fish oil (from herring), and dried carrot

GF1, grain-free diet 1; GF2, grain free diet 2; CB, cereal-based diet.

**Table 2:** Means and standard deviation of the chemical composition of tested diets (% as it is).

	GF1	GF2	CB
Crude protein	36.3 ± 0.21	31.6 ± 0.06	31.7 ± 0.25
Total fat	18.5 ± 0.30	19.3 ± 0.23	19.4 ± 0.81
Crude fiber	2.30 ± 0.10	2.37 ± 0.06	2.47 ± 0.12
TDF	7.68 ± 0.08	8.37 ± 0.05	9.43 ± 0.10
IDF	3.02 ± 0.02	3.76 ± 0.07	3.48 ± 0.08
SDF	4.66 ± 0.01	4.91 ± 0.03	5.95 ± 0.04
Ash	6.50 ± 0.30	6.00 ± 0.06	6.03 ± 0.17
Starch	25.0 ± 0.06	28.0 ± 0.02	27.9 ± 0.58
ME^*^	3990 ± 2.65	3990 ± 1.82	3997 ± 2.25

GF1, grain-free diet 1; GF2, grain-free diet 2; CB, cereal-based diet; IDF, Insoluble dietary fiber; SDF, soluble dietary fiber. ^*^ME, Metabolizable energy (kcal/kg), calculated according to the predictive equation indicated by NRC (3).

**Table 3:** Essential amino acids profile of the tested diets (means and standard deviation, % as it is).

Amino acid	GF1	GF2	CB
Arginine	2.11 ± 0.30	1.88 ± 0.26	1.80 ± 0.25
Histidine	0.76 ± 0.11	0.72 ± 0.10	0.68 ± 0.10
Isoleucine	1.13 ± 0.16	1.12 ± 0.16	0.97 ± 0.14
Leucine	2.32 ± 0.32	2.20 ± 0.31	2.07 ± 0.29
Lysine	2.08 ± 0.29	2.11 ± 0.30	1.65 ± 0.23
Phenylalanine	1.30 ± 0.18	1.22 ± 0.17	1.18 ± 0.17
Proline	2.20 ± 0.31	1.77 ± 0.25	2.21 ± 0.31
Threonine	1.27 ± 0.18	1.22 ± 0.17	1.08 ± 0.15
Tyrosine	0.87 ± 0.12	0.84 ± 0.12	0.77 ± 0.11
Valine	1.54 ± 0.22	1.48 ± 0.21	1.35 ± 0.19
Cysteine + Cistin	0.42 ± 0.06	0.37 ± 0.05	0.43 ± 0.06
Methionine	1.10 ± 0.15	1.00 ± 0.14	0.93 ± 0.13
Tryptophane	0.33 ± 0.03	0.33 ± 0.03	0.32 ± 0.03

GF1, grain-free diet 1; GF2, grain free diet 2; CB, cereal-based diet.

Dogs were fed to meet maintenance requirements (ME, kcal = 132 × BW^0.75^ kg; 3). Each diet was alternatively administered to all dogs for 50 days (15 of feeding adaptation and 35 of administration). In addition, during the experimental trial, the diets were adjusted according to the weight of the animals.

### 2.2. Clinical examination and blood sampling

Blood samples were collected (±10 mL) at recruitment and at the end of each nutritional phase in two tubes: one with EDTA, for blood count, and one with separator gel to obtain the serum for the biochemical profile. Whole blood samples intended for the evaluation of the blood count were refrigerated and quickly transported to the clinical analysis laboratory of the Department of Veterinary Medicine and Animal Production of the Federico II University of Naples. Each blood sample was analyzed using an impedance device to carry out an instrumental count (HeCo 5 Vet C, Real-Time Diagnostic Systems; San Giovanni a Valdarno, Italy) after slow and constant mixing for 20 min. At the kennel, to obtain the serum, the gel separator tubes were left at room temperature for approximately 15 min until the clot formed and then centrifuged for 10 min at a speed of 1,500 × *g*. The serum was stored at −80°C and subsequently sent on dry ice to a reference laboratory (Kornwestheim, Germany) where the following parameters were determined using a Beckman biochemical analyzer (Beckman Coulter AU5400; Olympus America, Melville, NY, United States): globulin, total protein (TP), albumin (Alb), alkaline phosphatase (AP), glutamic pyruvic transaminase (GPT), alanine transaminase (ALT), γ-glutamyl transferase (GGT), aspartate transferase (AST), glutamate dehydrogenase (GLDH), fructosamine (Fr), insulin, α-amylase, lipase (LP)l cholesterol (Col), triglycerides (Tri), creatinine (Crea), BUN, and creatine kinase (CK). Physical examination was conducted and the weight and the body condition score (BCS) of tested dogs were evaluated at the beginning of each experimental period. The blood count and biochemical profile at recruiment are reported in Tables 4, 5.

### 2.3. Postprandial glucose and insulin response tests

Blood samples were collected at 8:00 a.m. when dogs had been fasting for 12 h (baseline sample, time 0) to determine all the hematological parameters. In addition, blood samples were collected at 120, 240, and 360 min after the meal to measure dogs’ postprandial glycemic and insulin responses. The dogs received 50% of the ration after the first sampling (time 0) and the rest of the meal (50%) after the last sampling (360 min). Blood was collected at the beginning of each sampling (3 mL) in a Na-heparin tube, centrifuged (378 × *g* for 5 min), and the plasma was separated into two Eppendorf tubes. One drop of blood from the same sample was immediately used to measure glycemia using a portable digital glucometer (Sinocare Safe-Accu, Safecare Bio-tech, Yuhang, China). All the blood samples from the studied groups were obtained by an expert veterinarian to avoid possible mistakes during blood collection and measurement using the glucometer. Plasma samples were kept under refrigeration (4°C) for a maximum of 2 h before analysis. Insulin plasma samples were frozen (−80°C) for a maximum of 2 months before they were analyzed (11). Insulin was assessed by Chemiluminescence Enzyme Immunoassay (CLIA).

**Table 4:** Blood count of the tested dogs at recruitment.

Items	Units	Mean value	Reference value
RBC	M/μL	6.80 ± 0.64	5.50–7.90
WBC	K/μL	14.0 ± 3.02	6.00–16.0
Hgb	g/dL	16.2 ± 1.59	12.0–18.0
Hct	%	47.1 ± 4.53	37.5–55.0
MCV	fL	69.3 ± 2.53	60.0–76.0
MCH	Pg	23.9 ± 1.07	20.0–27.0
MCHC	g/dL	34.5 ± 0.44	32.0–38.0
Plt	K/μL	315 ± 87.7	240–400

RBC, red blood cells; WBC, white blood cells; Hgb, hemoglobin; Hct, hematocrit; MCV, medium corpuscular volume; MCH, medium corpuscular hemoglobin; MCHC, mean corpuscular hemoglobin concentration; Plt, platelets.

**Table 5:** Biochemical profile of the tested dogs at recruitment.

Items	Units	Mean value	Reference value
BUN	mmol/L	6.33 ± 2.06	3.2–10.3
Crea	μmol/L	79.8 ± 23.7	44–133
Tri	mmol/L	1.26 ± 0.77	0.3–5.3
Chol	mmol/L	4.14 ± 1.27	3.6–10.3
TP	g/L	63.0 ± 17.9	54–76
ALT	U/L	38.6 ± 14.7	25–122
Bil	μmol/L	2.52 ± 0.86	0–6.8
AP	U/L	43.8 ± 21.3	14–147
GGT	U/L	2.73 ± 1.46	2–13

Crea, Creatinine; Tri, Triglyceride; Chol, Cholesterol; TP, Total protein; ALT, Alanine Transaminase; Bil, Bilirubin; AP, Alkaline phosphatase; GGT, Gamma-glutamyl transferase.

### 2.4. Calculations

The integrated area under postprandial glucose and insulin response curves for each dog was calculated using the trapezoidal method (JMP 14, SAS Institute, NC, United States). Subsequently, the area of each dog was averaged to determine the AUC of each diet. In addition, based on the blood samples collected from each dog, the average concentration (mean concentration), maximum (zenith) and minimum (nadir) peaks, and the time to reach the maximum increase (time to peak) of glucose and insulin for each diet were determined.

### 2.5. Statistical analysis

The effect of diet was observed using a mixed model, in which time and animals were the random factors and the diet was the fixed factor. Tukey’s HSD test was used when significant differences were observed. All statistical analyses were performed using the software JMP 14 (SAS Institute, NC, United States).

## 3. Results

### 3.1. Biochemical profile

Table 6 shows the biochemical profile of tested dogs. During the trial, all parameters fell in the range indicated as physiological for the species (3). The highest level of albumin was registered when dogs were fed the GF2 diet, whereas the lowest levels were observed when the dogs were fed the GF1 diet. CB and GF2 diets resulted in the highest and lowest amounts of fructosamine, respectively (*p* < 0.05). Similarly, when the dogs were fed the cereal-based diet, they had the highest levels of glucose, whereas the GF1 group reported the lowest amount.

**Table 6:** Biochemical profile of the tested dogs.

Items	Units	GF1	GF2	CB	RMSE	Reference values
Gl	g/L	36.0	36.4	36.7	3.40	24–43
TP	g/L	66.4	67.1	66.0	3.65	54–76
Alb	g/L	28.6^b^	30.2^a^	29.3^ab^	1.14	28–43
AP	U/L	32.6	33.7	35.7	9.02	14–147
Crea	μmol/L	76.1	79.9	80.3	9.01	44–133
BUN	mmol/L	6.19	6.48	6.54	1.40	3.2–10.3
CK	U/L	126	112	114	50.2	41–378
ALT	U/L	45.6	43.3	44.9	12.9	25–122
GGT	U/L	3.12	2.96	3.34	0.95	2–13
AST	U/L	32.3	32.5	34.2	4.84	14–59
Fr	μmol/L	196^ab^	193^b^	206^a^	11.6	177–314
Glu	mmol/L	4.63^b^	4.86^ab^	4.97^a^	0.42	3.2–7.0
α-amylase	U/L	781	783	779	101	333–1264
LP	U/L	86.4	87.4	90.2	18.7	0.1–250
Chol	mmol/L	4.85	4.72	5.10	0.77	3.6–10.3
Tri	mmol/L	0.62	0.71	0.71	0.32	0.3–5.3
Bil	μmol/L	3.33	3.05	3.25	0.56	0.0–6.8

GF1, grain-free diet 1; GF2, grain-free diet 2; CB, cereal-based diet; Gl, Globulin; PT, Total protein; Alb, Albumin; AP, Alkaline phosphatase; Crea, Creatinine; CK, Creatine kinase; ALT, Alanine Transaminase; GGT, Gamma-glutamyl transferase; AST, Aspartate Transferase; Fr, Fructosamine; Glu, Glucose; LP, Lipase; Chol, Cholesterol; Tri, Triglyceride; Bil, Bilirubin. Along the row, lowercase letters indicate differences for *p* < 0.05. RMSE, root means square error.

### 3.2. Glucose and insulin postprandial responses

Table 7 shows the variation of serum glucose recorded during the trial. The GF1 diet showed the lowest (*p* < 0.001) values of AUC and the lowest (*p* < 0.05) nadir peak compared to the other diets. The mean glucose concentration was significantly lower (*p* < 0.01) in GF2 diet. The use of the CB diet showed the highest (*p* < 0.001) AUC values related to glycemia.

**Table 7:** Postprandial glucose response in the tested dogs.

Diet	GF1	GF2	CB	RMSE
Glucose (mg/dL)				
AUC 0–360 min	34236^B^	36371^A^	36406^A^	909
Mean concentration	87.3^AB^	82.8^B^	89.3^A^	4.26
Zenith	91.2	92.4	92.5	4.13
Nadir	76.7^b^	86.1^a^	82.0^ab^	5.52
Time to peak (min)	221	221	220	158

GF1, grain-free diet 1; GF2, grain-free diet 2; CB, cereal-based diet; AUC, area under the curve; Mean Concentration, medium level of serum glucose; Zenith, maximum level of glucose; Nadir, lowest level of glucose. Along the row, capital letters indicate *p* < 0.01, and lowercase letters indicate *p* < 0.05. RMSE, root mean square error.

Table 8 shows the trend of insulin response in the function of the administered diet. CB diet reported the highest insulin response in terms of AUC and mean concentration (*p* < 0.01). The cereal-based (CB) diet had the lowest (*p* < 0.01) zenith and nadir insulin levels (p < 0.01), and time at peak append earlier in comparison to the other diets (*p* < 0.05). Compared to other diets, the GF2 diet presented the highest (*p* < 0.001) zenith insulin level.

**Table 8:** Postprandial insulin response in the tested dogs.

Diet	GF1	GF2	CB	RMSE
Insulin (mIU/L)				
AUC 0–360 min	3550^B^	3674^B^	3897^A^	186
Mean concentration	9.73^B^	9.44^B^	11.9^A^	1.83
Zenith	14.7^B^	18.1^A^	13.1^B^	2.52
Nadir	9.80^A^	9.22^A^	6.45^B^	1.68
Time to peak (min)	316^a^	251^ab^	168^b^	109

GF1, grain-free diet 1; GF2, grain-free diet 2; CB, cereal-based diet; AUC, area under the curve; Mean Concentration, medium level of serum insulin; Zenith, maximum level of insulin; Nadir, lowest level of insulin. Along the row, capital letters indicate *p* < 0.01, and lowercase letters indicate *p* < 0.05. RMSE, root means square error.

**Figure 2:**
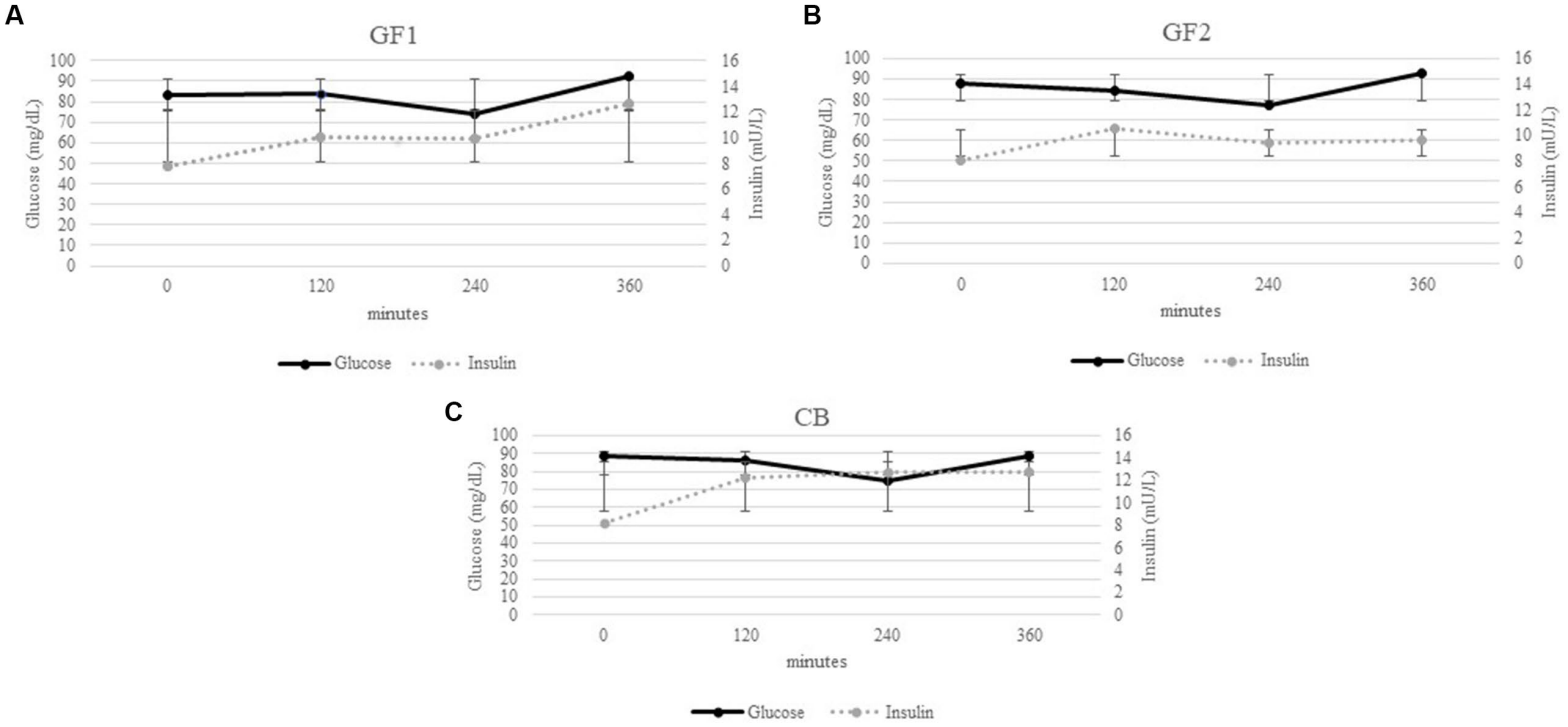
Glucose (mg/dL), Insulin (mL/UL).

Figures 2A–C describe the glucose and insulin postprandial curves obtained when dogs were fed the GF1, GF2, and CB diets, respectively. With diet GF1, the level of glucose was always higher than the insulin concentration. Nevertheless, after 360 min both concentrations seemed to be overlapping. Insulin concentration with the GF2 diet was greater than glucose concentration after 120 min. However, at 240 min glucose level increased compared to insulin concentration. With the CB diet, the glucose concentration was always greater than the insulin level, except at 240 min.

## 4. Discussion

Considering the nutritional characteristics, all the tested diets satisfied the nutritional requirements of adult dogs placed in a kennel (3). No refusals were observed during the experimental period, meaning that the diets were palatable. Furthermore, the amount of feed administered was correctly calculated during the trial, considering that no significant differences were observed regarding live weight and body condition scores.

### 4.1. Blood metabolic profile

All biochemical parameters fell into the physiological range for canine species (12). In our study, the highest levels of albumin and lowest concentration of fructosamine were reported in the dogs that were fed the GF2 diet. Furthermore, the dogs registered the lowest level of glucose and fructosamine when GF1 and GF2 diets were administered, respectively. These results were unexpected and could be related to the relatively high variability recorded for these parameters (13).

Serum proteins, such as albumin, act as important carrier substances and contribute to the regulation of acid–base balance. Moreover, the body’s immune system depends on protein substances (14). Serum fructosamine and plasma glucose are frequently used to assist in the diagnosis and monitoring of diabetes mellitus (15). The term fructosamine is a result of a nonenzymatic chemical reaction between a molecule of glucose and a free amino group (16). Furthermore, serum fructosamine reflects the degree of glycation of serum proteins and the mean serum glucose concentration from the previous 1–3 weeks in dogs, so it could be considered a longer-term marker of glycemic control in comparison with serum glucose measurement, which is a short-term marker (17). Moreover, the same authors observed that the serum concentration of fructosamine is not affected by acute increases in blood glucose concentration, which occur with glucose during stress or excitation.

### 4.2. Glycemic and insulin response

The postprandial glycemic response shows changes in blood glucose concerning different carbohydrate-containing foods (18). The interpretation of postprandial glycemic responses depends on several factors, such as ingested amount, processing, and diet composition (9). The amount of starch consumed and digested is one of the major factors that affects glucose response to the meal. In our study, the amount of starch intake was quite similar between the diets (75, 80, and 78 g/d for the GF1, GF2, and CB diets, respectively), suggesting a role of starch source on the glycemic and insulin responses. Carciofi et al. (5) investigated the effects of different starch sources, observing that extruded diets composed of similar ingredients but different starch sources can reveal important differences in postprandial glycemic response. Similarly, in the present study, we tested the use of three diets with similar nutritional characteristics but formulated with different carbohydrate sources (cereals grain vs. sweet potatoes vs. pea starch). However, it is difficult to compare our data with the literature due to some limitations in the experimental design (feed administration and sampling). The obtained results suggest that specific characteristics of these ingredients and their level of inclusion affected glycemic response (19, 20). The GF1 diet always reported the lowest values of glucose and insulin AUC. This result could be ascribed both to the lower starch amount of this diet (25 vs. 28% in the GF1 and the other diets, respectively) and to the digestibility of sweet potatoes, which were the main source of starch in the GF1 diet.

Carbohydrate sources, such as potatoes, sweet potatoes, peas, chickpeas, or lentils, are often used in pet foods. These ingredients also provide plant-based protein (21). *In vitro* studies have shown that sweet potatoes can result in a lower glycemic index (GI) due to their higher fibrous fraction and the higher proportion of amylose and resistant starch (RS), which may slow gastric emptying and reduce glucose absorption rate (22, 23). The term resistant starch (RS) indicated the starch residue left after hydrolyzing starch first with sulfuric acid (2 M) and then by incubating the residue with *α*-amylase and pullulanase (4, 18). Furthermore, the high amount of amylose appears to lower the rate of glucose delivery to blood, promoting a lower glycemic index (24).

Dogs that received the GF2 diet showed the highest glucose and lowest insulin AUC. In the GF2 diet, the main source of starch was pea starch. Pea starch is mainly available as a by-product of protein extraction. In our study, pea starch derived from wrinkled peas, which is more susceptible to be attacked by *α*-amylase. Furthermore, starch purification process often leads to changes in the starch structure and improves digestibility (25). In addition, thermal processing significantly increases the rapidly digestible starch and decreases the resistant starch fractions in pea starch (26). In this regard, legume starches are more digestible than potato starches, which are rich in amylose but less digestible than starches of several cereal grains (26). As reported by Yang et al. (27), amylopectin is more easily digested than amylose because amylopectin polymers have more intramolecular hydrogen bonds and less surface area. These characteristics could explain the glycemic and insulin responses of GF2 diet. Similarly, GF2 and CB diets showed higher glycemic responses. Whereas GF1 and GF2 diets registered similar insulin responses between the groups.

The observed digestion pattern could be related to the ingredients used in the formulation and the raw material processing method. As reported by Ottoboni et al. (7) and Giuberti et al. (28), technological treatments could cause starch gelatinization (not measured in the present work), which affects glucose release. In our case, some raw materials were heat-treated prior to the extrusion process and then subjected to double heat treatment.

The CB diet showed the highest glucose and insulin AUC and the lowest nadir and zenith values. These results could be due to the high proportion of whole spelt and oats (20%) in the diet, which allows faster energy availability compared to sweet potatoes and pea starch, as suggested by the lower time to peak. The results obtained when the dogs were fed the CB diet suggested a rapid digestibility of that diet. As suggested by Monti et al. (29), as faster and more complete the digestion and absorption of starch are, the greater the postprandial responses. However, compared to GF1 and GF2 diets, the CB diet is richer in total and soluble dietary fiber being composed by whole oat and spelt (30). Brennan and Clearly (31) reported the beneficial effects of soluble fibers on health. Cereal-based diets play a role in modulating the glucose absorption period and in lessening the variation in glucose and insulin concentrations (32). All these considerations were confirmed by the glycemic curve of three diets (Figures 1, 2A,B). In particular, a slower insulin response was observed in the GF1 curves when compared to the GF2 and CB ones. Moreover, the differences registered among the diets could be ascribed to differences in crude protein, total dietary fiber, ether extract, and starch. Indeed, all these nutrients could contribute to the plasma glucose and insulin response (33).

## 5. Conclusion

In recent years, owners of companion animals have been paying ever more attention to the nutrition and health of their animals. In this respect, the choice of the right diet is crucial regarding several factors, such as age and body weight. Despite some limitations in the experimental design, the obtained results show how different starch sources can lead to a different glycemic response. The grain-free diets (GF1 and GF2), even reported the lack of cereals, showed a different glycemic and insulin response due to the different starch digestibility. Whereas the CB diet showed an increase in glycemic response probably due to the rapid absorption of starch. Further studies are needed on the starch characteristics of tested diets and the potential benefits of these carbohydrates to dog health.

## Data availability statement

The raw data supporting the conclusions of this article will be made available by the authors, without undue reservation.

## Ethics statement

The animal study was reviewed and approved by the Ethics Committee for the care and use of animals of the University of Naples Federico II in accordance with local and national regulations and guidelines (Legislative Decree 26 of 04/03/2014).

## Author contributions

MG, MC, and SC contributed to the conception and design of the study. AV performed the statistical analysis. AV, AR, MA, and MF performed the analysis. AV, MG, and AR wrote the first draft of the manuscript. AR and MA wrote sections of the manuscript. All authors contributed to the manuscript revision and read and approved the submitted version.

## Funding

This trial was partially supported by Farmina Pet Food (Nola, Italia) and by funding from the DMVPA.

## Conflict of interest

The authors declare that the research was conducted in the absence of any commercial or financial relationships that could be construed as a potential conflict of interest.

## Publisher’s note

All claims expressed in this article are solely those of the authors and do not necessarily represent those of their affiliated organizations, or those of the publisher, the editors and the reviewers. Any product that may be evaluated in this article, or claim that may be made by its manufacturer, is not guaranteed or endorsed by the publisher.
